# Antidotal Potency of the Novel, Structurally Different Adsorbents in Rats Acutely Intoxicated with the T-2 Toxin

**DOI:** 10.3390/toxins12100643

**Published:** 2020-10-05

**Authors:** Vesna Jaćević, Jelena Dumanović, Miodrag Lazarević, Eugenie Nepovimova, Radmila Resanović, Zoran Milovanović, Qinghua Wu, Kamil Kuča

**Affiliations:** 1 Department for Experimental Toxicology and Pharmacology, National Poison Control Centre, Military Medical Academy, 11000 Belgrade, Serbia; 2 Department of Pharmacological Science, Medical Faculty of the Military Medical Academy, University of Defence, 11000 Belgrade, Serbia; dumanovicjelena@gmail.com; 3 Department of Chemistry, Faculty of Science, University of Hradec Kralove, Rokitanského 62 500 03 Hradec Králové, Czech Republic; evzenie.n@seznam.cz (E.N.); wqh212@hotmail.com (Q.W.); 4 Department of Analytical Chemistry, Faculty of Chemistry, University of Belgrade, 11158 Belgrade, Serbia; 5 Department for Physiology, Faculty of Veterinary Medicine, University of Belgrade, 11000 Belgrade, Serbia; lazarevicm@vet.bg.ac.rs; 6 Institute for Poultry Disease, Faculty of Veterinary Medicine, University of Belgrade, 11000 Belgrade, Serbia; radar@vet.bg.ac.rs; 7 Special Police Unit, Police Department of the City of Belgrade, Ministry of Interior, 11030 Belgrade, Serbia; tinahoks41@gmail.com; 8 College of Life Science, Yangtze University, Jingzhou, Hubei 434023, China

**Keywords:** T-2 toxin, adsorbents, rats, antidote

## Abstract

In this paper, the potential antidote efficacy of commercially available formulations of various feed additives such as Minazel-Plus^®^, Mycosorb^®^, and Mycofix^®^ was considered by recording their incidence on general health, body weight, and food and water intake, as well as through histopathology and semiquantitative analysis of gastric alterations in Wistar rats treated with the T-2 toxin in a single-dose regimen of 1.67 mg/kg p.o. (1 LD_50_) for 4 weeks. As an organic adsorbent, Mycosorb^®^ successfully antagonized acute lethal incidence of the T-2 toxin (protective index (PI) = 2.25; *p* < 0.05 vs. T-2 toxin), and had adverse effects on body weight gain as well as food and water intake during the research (*p* < 0.001). However, the protective efficacy of the other two food additives was significantly lower (*p* < 0.05). Treatment with Mycosorb^®^ significantly reduced the severity of gastric damage, which was not the case when the other two adsorbents were used. Our results suggest that Mycosorb^®^ is a much better adsorbent for preventing the adverse impact of the T-2 toxin as well as its toxic metabolites compared with Minazel-plus^®^ or Mycofix-plus^®^, and it almost completely suppresses its acute toxic effects and cytotoxic potential on the gastric epithelial, glandular, and vascular endothelial cells.

## 1. Introduction

T-2 mycotoxin is a particularly toxic natural metabolite of various fungi from the genus *Fusarium* [1,2,3]. Under specific storage conditions, namely a high temperature and high humidity, these moldy species may infect the majority cereal grains [4,5,6,7]. The oral intake of naturally infected food and feed with various toxic parts of *Fusarium* fungi can result in severe mycotoxicosis with signs of intoxication similar to T-2 toxin effects [8,9,10,11,12]. In humans and animals, the T-2 toxin causes general signs of the so-called shock-like syndrome, accompanied with emesis, lowered weight gain, lethargy, bloody diarrhea, gastric and intestinal disorders, haemorrhaging, immunosuppression, cardiomyopathy, and finally death [13,14,15,16,17,18]. Furthermore, similarly to acute radiation syndrome [19,20,21], inflammatory reaction, caused by the T-2 toxin, is a consequence of phospholipase A2 activation, successive prostaglandins, and reactive oxygen species (ROS) production [15,22,23]. Intensive accumulation of ROS may lead to DNA damage, protein oxidation, and lipid peroxidation [24,25,26]. Consequently, if lipid peroxidation is progressive, free radical generation and neutrophils infiltration of gastric mucosal tissue are tangled within the progression of acute gastric lesions [9,27,28]. As the T-2 toxin is extremely cytotoxic, it can break the conventional gastrointestinal entity both in vitro and in vivo [29]. There is a strong belief that T-2 toxic effects are a consequence of certain subcellular changes [4,5,30,31]. Thus, during a histopathology examination of the gastrointestinal tissue of rats, prominent mucosal lesions in an exceeding sort of petechiae and ulcerations were determined, presumably because of augmented capillary permeability and hemorrhages, and therefore a build-up of cells involved in inflammation was noted [32,33,34].

As mentioned earlier, these intensive gastric injuries are not only thanks to a local toxic effect [9,20], but to biliary excretion and enterohepatic recirculation of the T-2 and HT-2 toxins as well, [15,21] causing generalized toxic effects [34]. By providing the previously demonstrated mechanism of gastric toxicity induced by the T-2 toxin, an approach that supports the use of adsorbents seems to be rational. Numerous studies have shown that treatment with different adsorbents in clinical practice leads to a significant reduction and/or elimination of the adverse effects of varied mycotoxins [35,36,37,38,39,40]. In those studies, adsorbents, as substances unlikely to be absorbed from the intestine, have an exquisite potential to bind natural chemicals to themselves, thus preventing their absorption. To date, an outsized number of adsorbents, like activated charcoal, esterified glucomannan, various clays, and aluminosilicate zeolite, have shown significant protective effects against T-2 toxin acute intoxication, both in experimental studies and in clinical practice [41,42,43]. It has been shown that these inorganic adsorbents, as additives for the decontamination of food or feed, show selective binding of T-2 mycotoxins, preventing the passage of this toxin through the alimentary tract without adverse effects on general health, as well as its distribution in animal’s tissues or organs, which are used as edible nutrients for the human population. Moreover, inorganic adsorbents can fully bind aflatoxins, and are principally proposed to reduce their toxic effects [35,36,39,40]. During recent years, specific treatment using organic feed additives, such as modified mannan oligosaccharides, originating from the yeast cell wall inner layer, have been examined. Essentially, biological detoxification of the T-2 toxin is perhaps facilitated by the activity of the microorganism’s whole cells or the certain enzymes of only one cell [44].

Considering the above-mentioned findings, we have conducted a comprehensive study to gauge the potential antidotal efficacy of the commercially available formulation of the three various feed additives, namely Minazel-Plus^®^ (an inorganic-modified clinoptilolite), Mycosorb^®^ (an organic-esterified glucomannan), and Mycofix^®^ (a combined), by recording their incidence on general health, body weight, and food and water intake, and through histopathology and semiquantitative analysis of gastric alterations in Wistar rats acutely poisoned by the T-2 toxin.

## 2. Results

### 2.1. The Experimental Animals’ General Condition

Characteristic clinical symptoms of serious intoxication (i.e., vomiting, emesis, feed refusal, diarrhea, decreased body surface temperature, lethargy, and weakness) were only noticed in the group of animals treated with the T-2 toxin over a period of 2 to 4 days. Thereafter, no significant changes in the general health condition were observed in the surviving animals over the 28-day observation period.

Moreover, the absence of serious behavioral and gastrointestinal disorders was observed in the surviving rats from the groups protected with Minazel-Plus^®^ (MP), Mycosorb^®^ (MS), and Mycofix-plus^®^ (MF) over the 28-day observation period.

Moreover, these animals had been in a proper health condition, without any visible changes to the skin and mucosal membranes.

### 2.2. The Influence of Various Adsorbents on the Survival Rate in the T-2 Toxin-Treated Animals

By monitoring the survival rate after 24 h, we found that all of the applied adsorbents successfully antagonized the lethal effects of the T-2 toxin, but only at the highest dose (1.0 g/kg).

As presented in Table 1, the best protective index was accomplished with Mycosorb^®^ (2.25), with a value significantly higher than 1.31 and 1.79 rendered following treatment with the same total single dose of Minazel-Plus^®^ and Mycofix-plus^®^, respectively.

These protective effects established after 24 h remain at the same level throughout the 28 days of the experiment.

**Figure 1:**
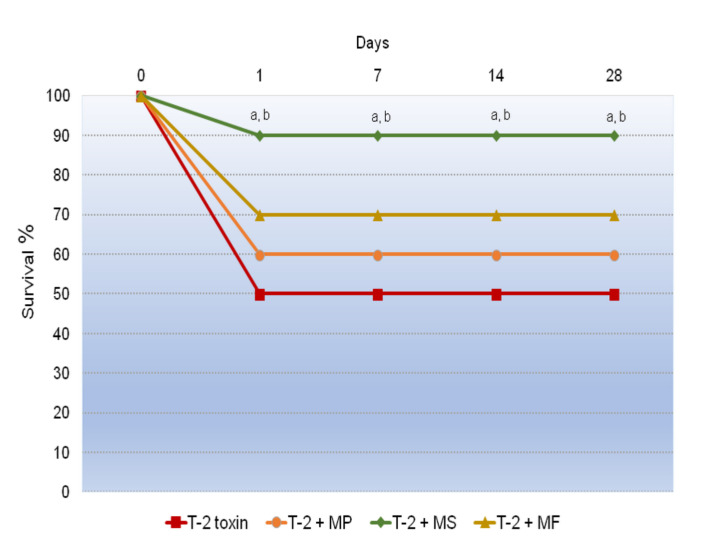
Time-dependent effects of Minazel-Plus^®^ (MP), Mycosorb^®^ (MS), and Mycofix-plus^®^ (MF) on the survival rates in T-2 toxin-treated rats: (a) *p* < 0.05 vs. Minazel-Plus^®^ (MP); (b) *p* < 0.05 vs. Mycofix-plus^®^.

In this part of the study, the comparative protective efficacy of Minazel-Plus^®^ (MP), Micosorb^®^ (MS), and Mycofix-plus^®^ (MF) was also examined. All of the adsorbents given at a dose of 1.0 g/kg resulted in good protection. A single dose of Minazel-Plus^®^ (MP) and Micofix-plus^®^ (MF) provided a survival rate of 65% and 75%, respectively, 24 h after T-2 toxin intoxication, while the survival rate of Micosorb^®^ (MS) animals was 90%. There was no statistically significant difference between the groups treated with Minazel-Plus^®^ (MP) and Mycofix-plus^®^ (MF). Moreover, the survival rates showed a statistical significance between Micosorb^®^ (MS) and Minazel-Plus^®^ (MP), as well as between Micosorb^®^ (MS) and Mycofix-plus^®^ (MF), throughout the study period (*p* < 0.5; Figure 1).

### 2.3. The Influence of Various Adsorbents on Body Weight Gain in T-2 Toxin-Treated Animals

**Figure 2:**
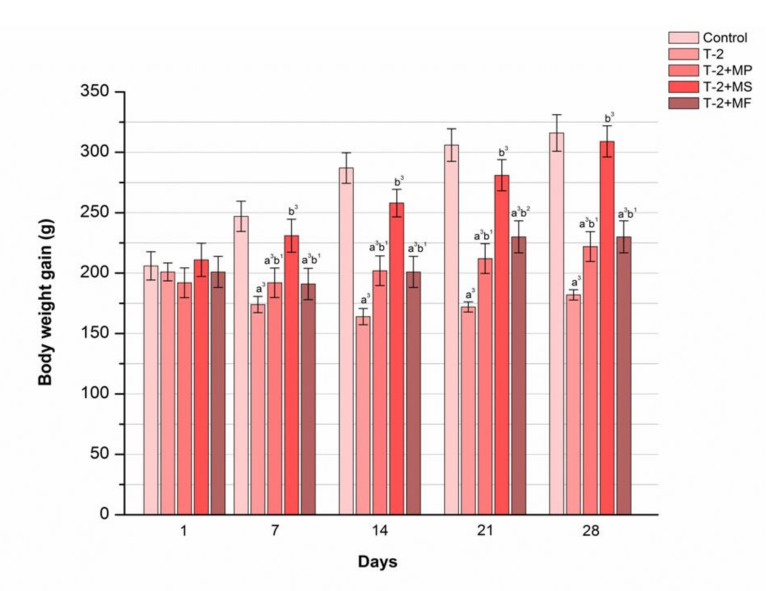
Time-dependent effects of Minazel-Plus^®^ (MP), Mycosorb^®^ (MS), and Mycofix-plus^®^ (MF) on body weight in T-2 toxin-treated rats (T-2): (a^3^) *p* < 0.001 vs. control; (b^1^, b^2^, b^3^) *p* < 0.05, 0.01, 0.001, respectively, vs. the T-2 toxin.

According to the data presented in Figure 2, the T-2 toxin induced a huge reduction in body weight compared with the control groups, with the lowest data being shown on the seventh day of this experiment (*p* < 0.001). Moreover, a mild increase in weight gain was registered from days 14 to 28, but it was significantly less than within the groups protected with Mycosorb^®^ (*p* < 0.001), Minazel-Plus^®^, and Mycofix-plus^®^ (*p* < 0.01). The best body weight was accomplished with Mycosorb^®^, and with values almost the same as for the control animals and notably above than those within the T-2 toxin-poisoned group (*p* < 0.001). Furthermore, a marked upgrowth of body mass was established within the Minazel-Plus^®^- and Mycofix-plus^®^-protected groups, with values significantly above those obtained within the unprotected group (*p* < 0.01). Moreover, body weight gain in these groups was significantly slower compared with the control group and Mycosorb^®^-treated group (*p* < 0.05).

### 2.4. The Influence of Various Adsorbents on Food Consumption in T-2 Toxin-Treated Animals

**Figure 3:**
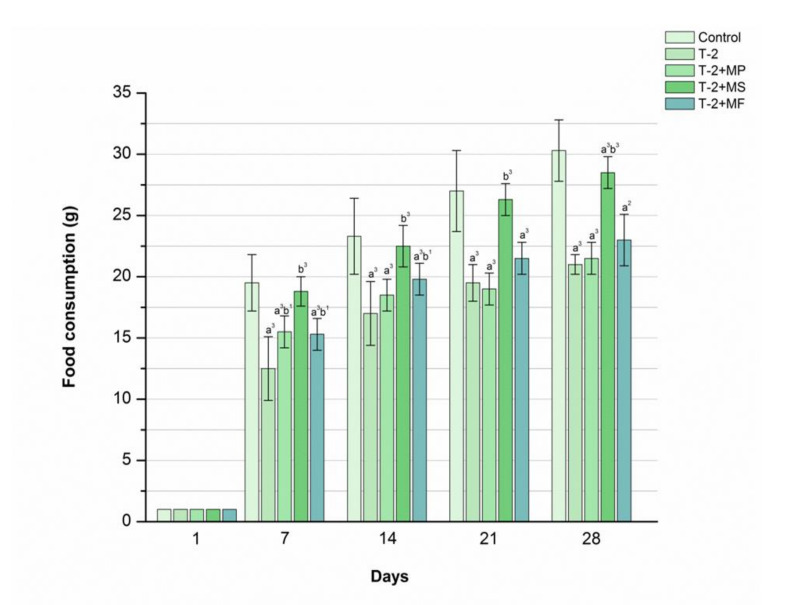
Time-dependent effects of Minazel-Plus^®^ (MP), Mycosorb^®^ (MS), and Mycofix-plus^®^ (MF) on food consumption in T-2 toxin-treated rats (T-2): (a^2^, a^3^) *p* < 0.01, 0.001, respectively, vs. control; (b^1^, b^3^) *p* < 0.05, 0.001, respectively, vs. T-2 toxin.

As presented in Figure 3, in the group of rats treated with only the T-2 toxin, a major reduction in food consumption was noticed one week after intoxication. Afterwards, a humble increase in food consumption was registered throughout the study period, with values significantly less than those for the control group (*p* < 0.001). On the contrary, the calculated values of food consumption in rats protected with Mycosorb^®^ were very almost the same as those within the control group of rats throughout the trial (*p* < 0.001). However, in the Minazel-Plus^®^- and Mycofix-plus^®^-treated group, a moderate increase in food consumption was registered during days 7 to 14; however, their values were slightly above those from the poisoned group of animals (*p* < 0.05). Hence, in these two experimental groups, by the end of the study, food consumption was almost the same in the T-2 toxin-treated groups.

### 2.5. The Influence of Various Adsorbents on Water Consumption in T-2 Toxin-Treated Animals

**Figure 4:**
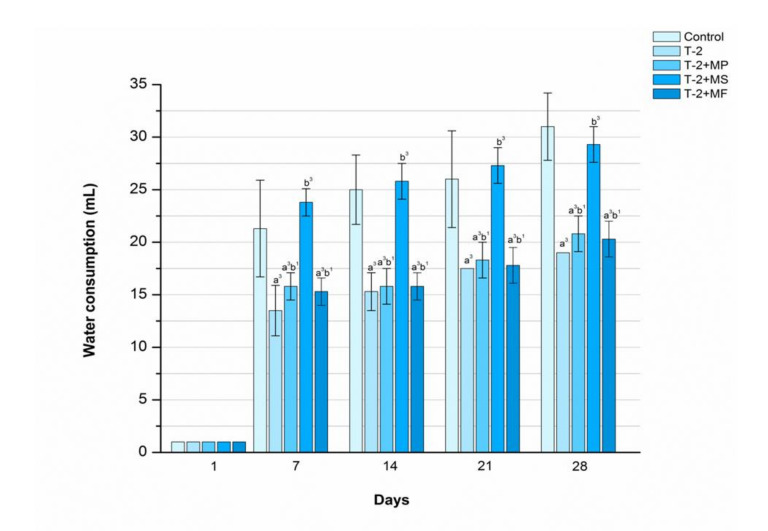
Time-dependent effects of Minazel-Plus^®^ (MP), Mycosorb^®^ (MS), and Mycofix-plus^®^ (MF) on water consumption in T-2 toxin-treated rats (T-2): (a^3^) *p* < 0.001 vs. control; (b^1^, b^3^) *p* < 0.05, 0.001, respectively, vs. T-2 toxin.

The results presented in Figure 4 clearly show that a complete single dose of Mycosorb^®^ initially significantly increased the quantity of consumed water compared with the poisoned animals (*p* < 0.001). Interestingly, these values were gently above those observed within the control group during the first three weeks of the study. Furthermore, treatment with Minazel-plus^®^ and Mycofix-plus^®^ also increased water consumption, but these differences were less pronounced (*p* < 0.05) in comparison with the poisoned and unprotected rats.

### 2.6. The Influence of Various Adsorbents on Gastric Damage of T-2 Toxin-Treated Animals

**Figure 5:**
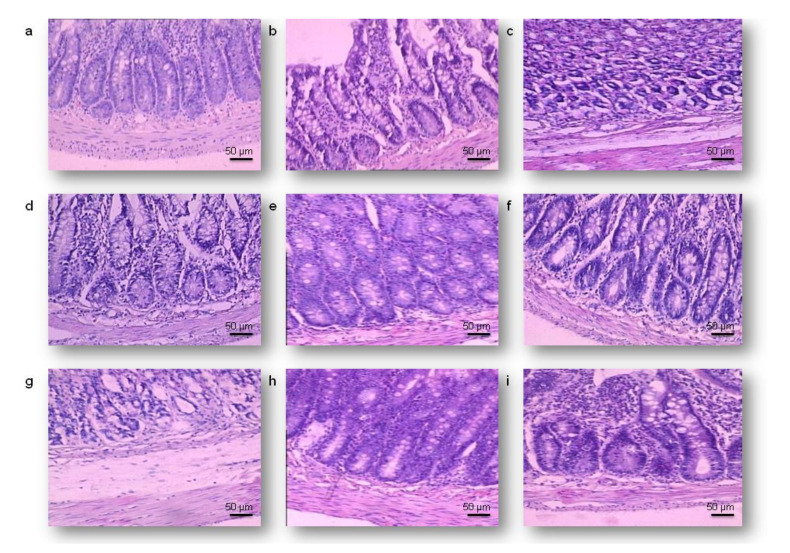
Gastric micrographs of rats treated with the T-2 toxin (T-2) and protected with Minazel-Plus^®^ (MP), Mycosorb^®^ (MS), and Mycofix-plus^®^ (MF); H&E staining, magnification 200×, scale bar = 50 µm: (**a**) control group, a gastric wall with no visible damage; (**b**) 7 days after treatment with T-2, ulceration in *the lamina epithelial*; (**c**) 28 days after treatment with T-2; cystic deformation of the gastric glands; (**d**) 7 days after treatment with T-2 + MP, erosions in the superficial epithelium; (**e**) 7 days after treatment with T-2 + MS, hyperemia and transmural oedema; (**f**) 7 days after treatment with T-2 + MF, degeneration of the epithelial and glandular cells; (**g**) 28 days after treatment with T-2 + MP, partial atrophy of the gastric pits; (**h**) 28 days after treatment with T-2 + MS, enlarged gastric pits; (**i**) 28 days after treatment with T-2 + MF, squamous degeneration of the epithelial cells.

The gastric tissue samples of the control rats had a histological structure without lesions (Figure 5a). After seven days of subsequent treatment, in the poisoned and unprotected animals, segmental degeneration, moderate hemorrhagic foci, and collections of neutrophils, macrophages, and mast cells (MCs) in *the tunica submucosa* were recorded (Figure 5b). The intensity of the described tissue alterations was increased on the 28th day of treatment, and their appearance was noticed diffusely (Figure 5c). Furthermore, on the day 28 of the trial, the T-2 toxin was triggered with a prominent lack of epithelial layer, submucosal oedema, reduced gastric pits, cystically altered gastric glands, and a large collection of inflammatory cells. A single application of Minazel-Plus^®^, Mycosorb^®^, or Mycofix-plus^®^ visibly reduced the intensity of the gastric injuries, the extent of intestinal bleeding, and the prevalence of inflammatory cells after 7 days (Figure 5d–f). However, at the end of the research period, the mildest degenerative alterations, focal haemorrhages, and single accumulation of inflammatory cells were observed in the poisoned rats protected with Mycosorb^®^ (Figure 5h) when compared with the group treated with Minazel-plus^®^ (Figure 5g) or Mycofix-plus^®^ (Figure 5i).

### 2.7. A Semiquantitative Evaluation of the Influence of Various Asorbents on the Gastric Damage of T-2 Toxin-Treated Animals

A detailed semiquantitative analysis proved that Mycosorb^®^ reduces the enormous gastric cell damage that occurred in the poisoned animals throughout the 28 days of the trial (Table 2). In the protected rats, the gastric damage score (GDS) was significantly lower when compared with the poisoned group only, starting on the seventh day of examination (*p* < 0.05). Additionally, the established damage was significantly higher when compared with the control animals throughout the research (*p* < 0.05). Therefore, the gastric damage detected in the poisoned rats treated with Minazel-plus^®^ ranged from exfoliation of the superficial epithelium (GDS = 2.80, day 7) to pronounced ulcerations, haemorrhages, and inflammatory cells infiltrations (GDS = 4.20, day 28). Quite similar gastroprotective effects were seen within the poisoned group protected with Mycofix-plus^®^, and the GDS values were 2.70 and 3.20, respectively.

## 3. Discussion

Consistent with our previously published results, the T-2 toxin certainly leads to acute gastric mucosal damage [20,45]. The calculated LD_50_ value of 1.67 mg/kg p.o. was also in agreement with the data of other authors, verifying that its acute toxicity, depending on the animal species and application mode, was in an exceedingly wide range (i.e., 0.5–10.5 mg/kg) [3,10,46]. Additionally, when the T-2 toxin was administered orally, gastric damage occurred directly by toxic influence on the *tunica mucosa*, and partially by indirect excretion of its toxic metabolites from the hepatobiliary system [20,29,47].

In this regard, we found typical clinical signs of intoxication in rats like vomiting, emesis, food refusal, diarrhea, decreased body surface temperature, lethargy, and weakness after 4 days of T-2 toxin administration at a single dose of 1.67 mg/kg p.o. Namely, the described clinical signs exhibited by the T-2 toxin-poisoned animals were similar to the symptoms reported earlier in calves [48,49], sheep [50], pigs [51], and goats [34]. Typical symptoms of T-2 toxicosis in lambs were expressed in the form of recurrent diarrhea associated with infection of the *Eimeria* species [50]. Additionally, particular symptoms observed during T-2 toxin intoxication, like lethargy and feed refusal, could be due to impairment within the central nervous system [52]. Partial or complete feed refusal, prolonged bloody diarrhea, and decreased gastrointestinal absorptive capacity were also registered in cattle [53,54,55]. Because of the complete refusal of feed, reduction of protein synthesis, and consequent weight loss, many organs were damaged, primarily the kidneys and liver, as well as the gastrointestinal and lymphatic tissue [4,34]. As noted by other scientists [42,44], we also observed a complete absence of previously mentioned clinical signs of T-2 toxin intoxication following the therapeutic administration of Mycosorb^®^, Minazel-Plus^®^, and Mycofix-plus^®^ over the whole research period.

Furthermore, in our previous studies, Mycosorb^®^, Minazel-Plus^®^, and Mycofix-plus^®^, given in the same dose, showed a promising protective potential against the adverse influence of the T-2 toxin in rats [18,19,45]. Throughout this study, Mycosorb^®^ administered at the same dose of 1 g/kg p.o., shortly after treatment with the T-2 toxin, significantly reduced the LD_50_ value (Table 1), indicating that the applied dose was very safe and therapeutically effective only in case of T-2 poisoning. Moreover, Mycosorb^®^ had the highest protective index (PI = 2.25), which was well above the 1.31 and 1.79 obtained following treatment with Minazel-Plus^®^ and Mycofix-plus^®^, respectively. In this manner, Mycosorb^®^ showed a high capacity for suppressing the T-2 toxin’s lethal outcomes. A lack of similar effects after the same dose of Minazel-Plus^®^ and Mycofix-plus^®^ might be caused by the lower adsorptive properties within the gastrointestinal tract. This process could potentiate the direct local toxic effect of the T-2 toxin and its active metabolites after enterohepatic recirculation, thus disrupting the protective effects of Minazel-Plus^®^ and Mycofix-plus^®^. The lack of a full protective potential of Minazel-Plus^®^ and Mycofix-plus^®^ to completely prevent T-2 toxin intoxication may be explained by the fact that inorganic materials such as activated charcoal, clay, bentonite, and aluminosilicate (principally proposed to reduce the toxic effects of aflatoxins) [35,36,39,40], have a limited efficacy relative to other toxins [38]. Moreover, almost all inorganic adsorbents contain dioxin and heavy metals, which are toxic.

On the other hand, organic adsorbents like Mycosorb^®^ have consequently been recommended as an alternative solution to adsorb numerous mycotoxins, including the T-2 toxin, but without spoiling the nutrient bioavailability or inducing adverse health effects in animals [56]. In agreement with other researchers, we observed [34,37,42,44] a significant lowering of the rats’ body weight and food and water consumption from the first week after the application of T-2 toxin. Although the body mass and food and water consumption of the Minazel-Plus^®^- and Mycofix-plus^®^-treated rats were also markedly decreased in comparison with the control animals, it was still greater than in the group treated with the T-2 toxin only, especially during the last three weeks of the trial. When a relatively high single dose of the T-2 toxin was administered, a significant decrease in body weight and food and water consumption was observed compared with the control group [19,44]. This could be explained by the loss of appetite, complete feed refusal, and substantial lesions in the *tunica mucosa* of the gastrointestinal tract accompanied by prolonged bloody diarrhea, which prevented food and water intake and its utilization [45,54,55].

Furthermore, the T-2 toxin leads to segmental degeneration, moderate hemorrhagic foci, and infiltration of gastric mucosal inflammatory cells over seven days (3.5 ± 0.5). The overall number of gastric mucosal injuries accompanied by petechiae and ulcerations was slightly higher after 28 days (4.2 ± 0.4). As mentioned earlier, gastric mucosa alterations are not only generated because of direct local toxic effects [9,10,29], but are also a consequence of the T-2 toxin and its active metabolites’ biliary excretion and enterohepatic recirculation causing general toxic effects [9,15,47]. Moreover, a single treatment with Minazel-Plus^®^, Mycosorb^®^, and Mycofix-plus^®^ visibly reduced the intensity of the gastric mucosa injuries, hemorrhagic foci, and prevalence of inflammatory cells after seven days of the study (GDS = 1.5 ± 0.5, 2.7 ± 0.3, and 2.8 ± 0.4). It was shown that the mildest degenerative and vascular alterations, as well as inflammation of the cell’s foci, were observed in the poisoned rats protected with Mycosorb^®^ (2.1 ± 0.3) when compared with the group treated with Minazel-plus^®^ (3.5 ± 0.5) or Mycofix-plus^®^ (3.2 ± 0.4) at the end of the research period. These results support the claim that aluminosilicates, as dietary supplements, do not fully protect animals from the harmful effects of T-2 toxins [44,57,58]. Namely, aluminosilicates are generally able to absorb almost all aflatoxins, while in others with chemically different mycotoxins, they exhibited a partial or incomplete adsorption potential [59]. On the other hand, Mycofix-plus^®^, as a mixed feed additive, contains the enzyme de-epoxidase, which selectively binds to the 12,13-epoxy-trichothecene ring of the T-2 toxin, thereby initiating the deacetylation process and its transformation into a less toxic de-epoxy-HT-2 toxin, making it a more potent adsorbent compared with aluminosilicates [60]. Given together with the T-2 toxin, the organic adsorbent Mycosorb^®^ expressed a significant decrease in the occurrence of cytotoxic alteration for both gastric epithelial and glandular cells, as well as vascular endothelial cells, over the 4-week study period. The ligands, mainly β-D-glucans, derived from the yeast cell’s wall, expressed the most potent functional efficiency in forming a complex with various mycotoxins in naturally or artificially contaminated animal feed [56,61,62,63,64]. It is assumed that the protective role of the applied organic adsorbent is a consequence of generating a tight biological complex with the T-2 toxin or its active metabolites, suppressing their negative impact on the gastric mucosal cells, and thus alleviating the production of pro-inflammatory substances [9,17,18] and the further release of reactive oxygen species and cytokines (TNF-α, IL-1, and IL-6) [21,65,66] as crucial mediators of oxidative stress and further development of the inflammatory reaction.

## 4. Conclusions

These results suggest that Mycosorb^®^, compared with Minazel-plus^®^ or Mycofix-plus^®^, is a much better adsorbent for preventing the adverse impact of the T-2 toxin, as well as its toxic metabolites, and it almost completely suppress its acute toxic effects and cytotoxic potential on the gastric epithelial, glandular, and vascular endothelial cells.

## 5. Materials and Methods

### 5.1. Experimental Animals

In this trial we used 6–8 week old (200–220 g) adult Wistar rats, raised at the Institute for Biomedical Researches, Military Medical Academy, Belgrade, Serbia. A typical macrolon plastic cage (Bioscape, Germany) filled with clear sawdust (Versele-Laga, Deinze, Belgium) was used for housing the experimental animals. The animals were housed in centrally regulated ambient conditions, with a temperature of 22 ± 2 °C, humidity of 55 ± 15%, air changes/h of 15–20, and light/dark cycle of 12/12 h. A commercial diet mixture for rats (Veterinary Institute Subotica, Subotica, Serbia) and tap water were applied ad libitum. Before the start of the study, the experimental design, laboratory protocol, and welfare of the experimental animals were approved by the Ethics Committee of Experimental Animals of the Military Medical Academy, Belgrade, Serbia (no. 282-12/2002, date: 2 December 2002.). This decision confirmed that the complete experimental study, animal care, and all of the treatments throughout the research are in compliance with Directive 2010/63/EU for the protection of animals used for scientific purposes, as well as the Guidelines for Animal Welfare adopted by the Republic of Serbia (no. 323-07-04943/2014-05/1; date: 8 December 2014).

### 5.2. T-2 Toxin

The T-2 toxin was isolated from *Fusarium sporotrichoides* fungi (ITM-391, originated from Serbian cereals in an accredited laboratory (Center of the Bio-Ecology, Zrenjanin, Republic of Serbia) [67,68,69], as previously described [9].

The T-2 toxin was applied in a single total dose of 1.67 mg/kg p.o., as previously described [9,17,18,70].

### 5.3. Various Commercial Adsorbents

In this study, three commercially available formulations of the following adsorbents were used: (1) Minazel-plus^®^ (MP; Patent Komerc, Mišićevo, Serbia), as an inorganic adsorbent obtained by organic modification of natural zeolite; (2) Mycosorb^®^ (MS; Alltech, Geneva, IL, USA), organic adsorbent based on esterified glucomannan derived from Saccharomyces cerevisiae 1026; and (3) Mycofix-plus^®^ (MF; Biomin, Herzogenburg, Austria), a mixed multi-component adsorbent composed of microorganisms (i.e., genus novus of the family Coriobacteriaceae), enzymes, and plant phycophytes.

Directly before p.o. application, each adsorbent was dissolved in a commercially prepared NaCl solution (0.9%), extempore.

### 5.4. Protective Effects of Commercial Adsorbents

For the purpose of determining the protective effects of Minazel-plus^®^ (MP), Mycosorb^®^ (MS), and Mycofix-plus^®^ (MF), the animals were poisoned by applying increasing doses of the T-2 toxin p.o. (0.75, 1.5, and 2.25 mg/kg p.o.). Following p.o. application, increasing doses of each adsorbent were applied (0.5, 0.75, and 1.0 g/kg).

The mean lethal doses were calculated after 24 h [18,45], and afterwards, the protective indexes (PIs) were calculated according to the previously published equation of PI = LD_50_ (T-2 toxin + adsorbent)/LD_50_ (T-2 toxin) [9]. Survival was monitored over a period of 28 days after treatment. The percentage of survival for all of the poisoned and then protected animals was calculated [45].

As already shown [18,45], in the rats treated with increasing doses of the T-2 toxin, the best protective effects were achieved by applying the highest single dose of each adsorbent (1.0 g/kg p.o.). These protective effects established at 24 h remained at the same level throughout the 28 days of the experiment. Accordingly, the same dose of each adsorbent was chosen for the rest of this study.

### 5.5. Experimental Design

After randomization, animals from five experimental groups, with 16 animals per group, were treated as follows: (1) control (0.9% saline 1 mL/kg p.o.), (2) T-2 toxin (T-2, 1.67 mg/kg p.o.), (3) T-2 toxin (T-2, 1.67 mg/kg p.o.) and Minazel-plus^®^ (MP, 1 g/kg p.o.), (4) T-2 toxin (T-2, 1.67 mg/kg p.o.) and Mycosorb^®^ (MS, 1 g/kg p.o.), and (5) T-2 toxin (T-2, 1.67 mg/kg p.o.) and Mycofix-plus^®^ (MF, 1 g/kg p.o.). First, the experimental animals were treated with the T-2 toxin, and immediately afterwards they were administered the examined adsorbents.

Then, the antidotal efficacy of each adsorbent was assessed by recording its influence on general health, body weight, and food and water intake in the T-2 toxin treated rats, over the whole 4-week study period.

### 5.6. Histopathological Examination

In a separate experiment of the same design, the gastric tissues of four rats from each experimental group were taken 7 and 28 days after treatment, and were prepared for histopathological analysis.

Specifically, four surviving animals from each experimental group were sacrificed, through anaesthetic by light ether, and each of the gastric samples were fixed in neutral buffered formalin (10%) for 5 to 7 days. Thereafter, six tiny slices from each gastric sample were dehydrated using xylene, a graded alcohol solution, and they were placed into paraffin blocks.

Each 2-μm thick paraffin section was stained with hematoxylin and eosin (H&E), and exanimated at a magnification of 200× using a light microscope (BKS-45, Olympus, Japan), according to our previously published methodology [9,17,18,19,20,71,72,73,74,75,76,77].

### 5.7. Semiquantitative Analysis

By using a light microscope, the distinctive gastric damage was analyzed and counted in accordance with a five-point semiquantitative scale developed for the assessment of the gastric damage score—gastric damage score (GDS; Table 3).

### 5.8. Statistical Analysis

A careful statistical examination was performed using commercial statistical software (Stat for Windows, R.7, Stat Soft, Inc., Tulsa, OK, USA, 2008). In the tables, the results are presented as the mean (X) ± standard deviation (SD). In the figures, the comparisons of the obtained results for body weight and food and water intake were done with the Student’s *t*-test, with significant differences expressed as *p* < 0.05, *p* < 0.01, and *p* < 0.001. The one-way ANOVA and post-hock analysis (i.e., Tuckey’s test), with a level of statistical significance set at *p* < 0.05, were used to determine the differences in the severity of the gastric damage score.

## Figures and Tables

**Table 1:** The protective effects of Minazel-Plus^®^ (MP), Mycosorb^®^ (MS), and Mycofix-plus^®^ (MF) on the 24-h survival in T-2 toxin-treated rats.

Treatment	LD_50_	95% Confidence Limit	f(LD_50_)	Protective Index (PI)
T-2	1.67	1.27–2.19	1.31	-
T-2 + MP	2.18	1.77–2.88	1.36	1.31
T-2 + MS	3.75	3.08–4.42	1.35	2.25
T-2 + MF	2.98	2.28–3.52	1.51	1.79

LD_50_ was calculated according to Litchfield and Wilcoxon. Protective indexes were calculated according to the following equation: PI = LD_50_ (T-2 + adsorbent)/LD_50_ (T-2).

**Table 2:** The protective effects of Minazel-Plus^®^ (MP), Mycosorb^®^ (MS), and Mycofix-plus^®^ (MF) on the severity of T-2 toxin-induced gastric changes in rats—gastric damage score (GDS).

Treatments	GDS (Mean Number (X ± SD) (4 Stomachs/Group × 8 Slices/Stomach))	
	7th day	28th day
Control	0.2 ± 0.4	0.2 ± 0.4
T-2	3.5 ± 0.5 ^a^	4.2 ± 0.4 ^a^
T-2 + MP	2.8 ± 0.4 ^a^	3.5 ± 0.5 ^a^
T-2 + MS	1.5 ± 0.5 ^a,b^	2.1 ± 0.3 ^a,b^
T-2 + MF	2.7 ± 0.3 ^a^	3.2 ± 0.4 ^a^

Tuckey’s test was applied for statistical analysis; (a) *p* < 0.05 vs. control, (b) *p* < 0.05 vs. T-2.

**Table 3:** Histopathological scoring scale for gastric damage in treated rats—gastric damage score (GDS).

Grade	Definition
0	Normal histological structure of the stomach
1	Mild alteration: segmental loss of the superficial epithelium, discrete vasodilatation of the blood vessels, and single inflammatory cells
2	Moderate damage: exfoliation of the surface epithelium, strong vasodilatation of the blood vessels, and inflammatory cell infiltration
3	Severe, focal damage: erosion of the epithelium, hemorrhages, and inflammatory cell infiltration
4	Severe, diffuse damage: pronounced ulcerations, hemorrhages, and inflammatory cell infiltration
5	Tissue necrosis
